# Toward sustainable activated carbon from peanut shells for efficient cationic dye removal: equilibrium, kinetics, and cost evaluation

**DOI:** 10.1038/s41598-026-58076-y

**Published:** 2026-06-26

**Authors:** Aya S. Mahmoud, Mohamed El Saied, Seham Ali Shaban, Ahmed O. Abo El Naga

**Affiliations:** 1 Chemistry Department, Faculty of Women, Ain Shams University, Cairo, Egypt; 2 Refining Division, Egyptian Petroleum Research Institute, Nasr City, Cairo, 11727 Egypt

**Keywords:** Biomass-derived activated carbon, Phosphoric acid activation, Agricultural waste valorization, Adsorption kinetics, Cationic dye adsorption, Chemistry, Environmental sciences, Materials science

## Abstract

Transforming abundant agricultural residue into high-performance carbonaceous adsorbents has emerged as a green and cost-effective strategy for the efficient removal of dyes from contaminated effluents. In this study, activated carbon adsorbents (referred to as PNSAC-x) were synthesized from peanut shells (PNS) using phosphoric acid as a cost-effective activating agent. Activation was conducted at a relatively low temperature of 400 ℃, employing PNS: H_3_PO_4_ weight ratios of 1:1, 1:2, and 1:3. The developed carbons were evaluated for their adsorption performance in removing crystal violet (CV) as a model cationic dye. Among the synthesized samples, PNSAC-3 (1:3 ratio) exhibited superior performance, achieving 98.9% CV removal within only 20 min at natural pH, with an adsorbent dosage of 0.3 g L^− 1^ at 25 °C. Comprehensive characterization confirmed that PNSAC-3 possesses an amorphous structure with a well-developed porous network, reaching a maximum adsorption capacity of 145 mg g^-1^ under the investigated conditions. Cost analysis demonstrated the economic feasibility of PNSAC-3 production, with an estimated cost as low as 115 LE kg⁻¹ using commercial-grade phosphoric acid. The adsorption kinetics followed the pseudo-second-order model (R^2^ = 0.998), while the equilibrium data were best described by the Langmuir isotherm model (R^2^ = 0.999). Thermodynamic studies indicated a spontaneous and endothermic adsorption process with increased feasibility at elevated temperatures. pH-dependent studies showed that the adsorption process involved both electrostatic forces and non-electrostatic interactions, including hydrogen bonding, hydrophobic effects, and π-π interactions. PNSAC-3 also demonstrated excellent reusability, retaining more than 94.4% of its adsorption efficiency after five adsorption–desorption cycles. These findings underscore the potential of PNSAC-3 as a highly efficient and cost-competitive adsorbent for the large-scale removal of cationic dyes from industrial wastewater.

**Supplementary Information:**

The online version contains supplementary material available at 10.1038/s41598-026-58076-y.

## Introduction

Numerous industries use vast quantities of dyes, a substantial portion of which is unavoidably discarded into nearby aquatic bodies daily without adequate treatment^1,2^. Due to their high-water solubility, persistence, and toxicity, decontaminating dye-polluted effluents has become a global concern. This has sparked extensive research interest in environmental science over recent decades^2–4^. Accordingly, a wide range of physical, chemical, and biological treatment strategies has been developed for sequestering dyes from contaminated effluents^5^. These methods include coagulation, precipitation, flocculation, biological degradation, and adsorption. Among these, adsorption stands out as an eco-friendly and facile strategy, allowing the efficient capture of these contaminants from aqueous solutions at low cost and with minimal energy demands^3^.

To date, a wide range of porous solids has been appraised for their ability to decontaminate dye-polluted effluents through adsorption. These include activated carbon^3^ polymers^6^, biosorbents^7^, metal-organic frameworks^8^, and zeolites^9^. Among these, activated carbon (AC) holds a special place owing to its large specific surface area, adjustable porosity, availability of surface functional groups, and exceptional adsorption capacity for various organic dyes^10,11^. Biomass wastes are gaining attention as sustainable sources for AC production due to their widespread availability, renewable nature, biodegradability, affordability, and favorable environmental impact^12–14^. Biomass materials, such as rice husks, corn cobs, wood, orange peels, and sugarcane bagasse, have been successfully utilized to produce activated carbons with high adsorption capacities for organic dyes^12,15–18^. The chemical activation method has proven effective in producing high-quality activated carbons with high porosity and desirable surface characteristics at relatively low activation temperatures from diverse biomass sources^14^. Various chemical activators, such as ZnCl_2_, KOH, and H_3_PO_4_, have been used in this process^14,19,20^. Phosphoric acid is widely used as an activating agent due to its effectiveness in developing porous carbon structures at relatively low activation temperatures and its ability to produce high carbon yields with predominantly mesoporous structures^21–23^. However, certain environmental considerations should be acknowledged. The washing step after activation may generate phosphate-containing wastewater that requires appropriate treatment before discharge^24^. Additionally, phosphoric acid production from phosphate rock is associated with environmental impacts. Nevertheless, compared with alkaline activating agents such as KOH or NaOH, phosphoric acid enables lower activation temperatures and promotes efficient pore development, while avoiding heavy metal contamination risks associated with ZnCl_2_^24–26^. Additionally, the lower activation temperature allows the retention of more acidic functional groups, such as lactones, carboxyl groups, and phenolic hydroxyl groups, in the produced activated carbon^27–29^. Acid activation can be achieved through pre-treatment and post-treatment strategies. In pre-treatment activation, the biomass is impregnated with the activating agent prior to carbonization, whereas, in contrast, post-treatment involves modifying the already formed biochar. In this study, phosphoric acid impregnation was applied as a pre-treatment method due to its ability to enhance pore formation during carbonization and reduce overall energy requirements by integrating activation and carbonization into a single step^30,31^.

This study focuses on producing peanut shell-derived activated carbons (PNSACs) with a high specific surface area through phosphoric acid activation at 400 °C. Three samples were obtained using varying H_3_PO_4_/PNS weight ratios of 1:1, 1:2, and 1:3. It is hypothesized that phosphoric acid activation of peanut shell biomass at 400 °C will produce a highly porous activated carbon with enhanced surface area and surface functionalities, which will significantly improve its adsorption performance toward crystal violet dye in aqueous solutions. Peanut shells were chosen as the biomass feedstock due to their high carbon content and low ash levels^32–35^. Peanuts (Arachis hypogaea L.) are a major global crop cultivated in over 100 countries^36^. In Egypt, the cultivated peanut area reached approximately 14,267 feddans, with a total yield of 199,000 tons in the 2019 season^37^. The large quantities of shells generated annually from peanut processing have little or no economic value. Utilizing these shells for AC production not only provides an abundant, and affordable resource but also adds economic value to these by-products, thereby supporting environmental sustainability.

The performance of PNSAC was assessed for its effectiveness in removing crystal violet (CV) from aqueous solutions. Crystal violet (CV), also known as Gentian Violet, is a cationic triphenylmethane dye widely used as a colorant in the textile industry, an indicator in chemical analysis, and for biological staining^38^. Despite its extensive applications, CV has been reported as a recalcitrant pollutant that persists in aquatic environments for prolonged periods due to its high chemical stability^38^. In addition to its environmental persistence, CV exhibits severe toxicological effects, being classified as a mitotic poison, potent carcinogen, and clastogenic agent, with reported tumor-promoting effects in some aquatic organisms^38,39^. It is also associated with skin, eye, liver malfunction, renal failure, and allergies^40,41^, and is therefore considered a significant biohazard substance^39^. These combined properties make CV a representative and challenging model pollutant for evaluating adsorption performance in wastewater treatment studies. Various techniques were employed to gain insight into the structural and morphological features of the obtained activated carbon, including X-ray diffraction analysis (XRD), Fourier transform infrared spectroscopy (FTIR), N_2_ physisorption measurements, Raman spectroscopy, scanning electron microscopy (SEM), and transmission electron microscopy (TEM). A comprehensive cost analysis of PNSAC production from PNS through H_3_PO_4_ activation was carried out to assess its affordability and feasibility for practical adsorption-based water purification applications. The effects of different adsorption parameters, such as solution pH, contact time, adsorbent dose, and initial dye concentration, on the efficiency of CV dye adsorption were evaluated. Additionally, to obtain a deeper understanding of the adsorption mechanism, adsorption isotherm, and kinetics studies were conducted. Ultimately, the real-world applicability of the current adsorption process was assessed through a recyclability study.

## Experimental

### Chemicals

**Fig. 1:**
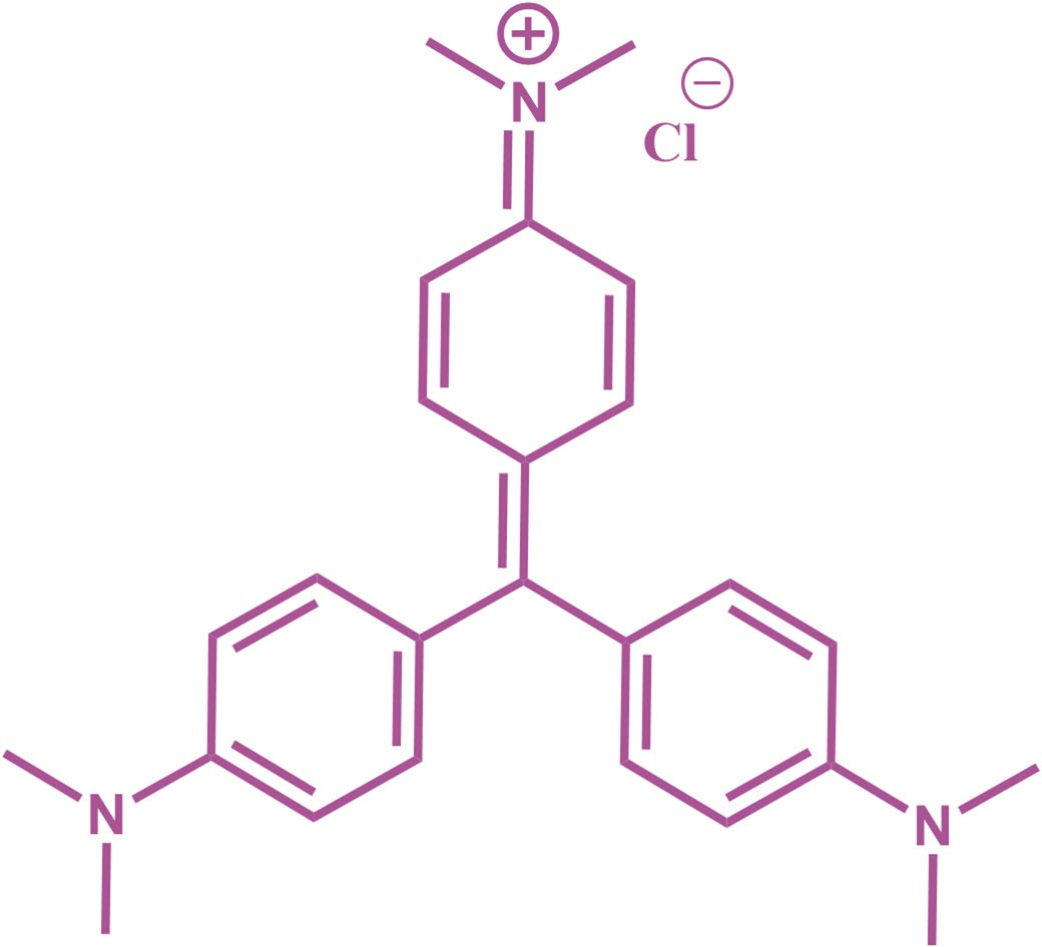
Molecular structure of CV.

Crystal Violet dye (CV; tris(4-(dimethylamino)phenyl)methylium chloride) was provided by Himedia Laboratories Pvt. Ltd. CV is a cationic triarylmethane dye with the Color Index (C.I.) number 42,555, a molecular formula of C_25_H_30_ClN_3_, and a molecular mass of 407.98 g/mol. The chemical formula of CV is provided in Fig. 1. CV has a maximum absorption wavelength of 584 nm. Stock solution with a CV content of 1000 mg L^-1^ was obtained by dissolving 1 g of CV dye in one liter of deionized water.

Peanut samples were sourced from a local market in Nasr City, Cairo, Egypt. The shells (PNS) were removed and thoroughly rinsed with water to eliminate impurities before being oven-dried. Afterward, the cleaned shells were milled to a particle size of less than 2 mm using a household chopper, and the resulting powder was kept for future use.

Hydrochloric acid (HCl) and sodium hydroxide (NaOH) were obtained from Sigma-Aldrich, while acetic acid and Phosphoric acid (85%, analytical grade) were purchased from ADWIC and Merck, respectively.

### Activated carbon production

**Fig. 2:**
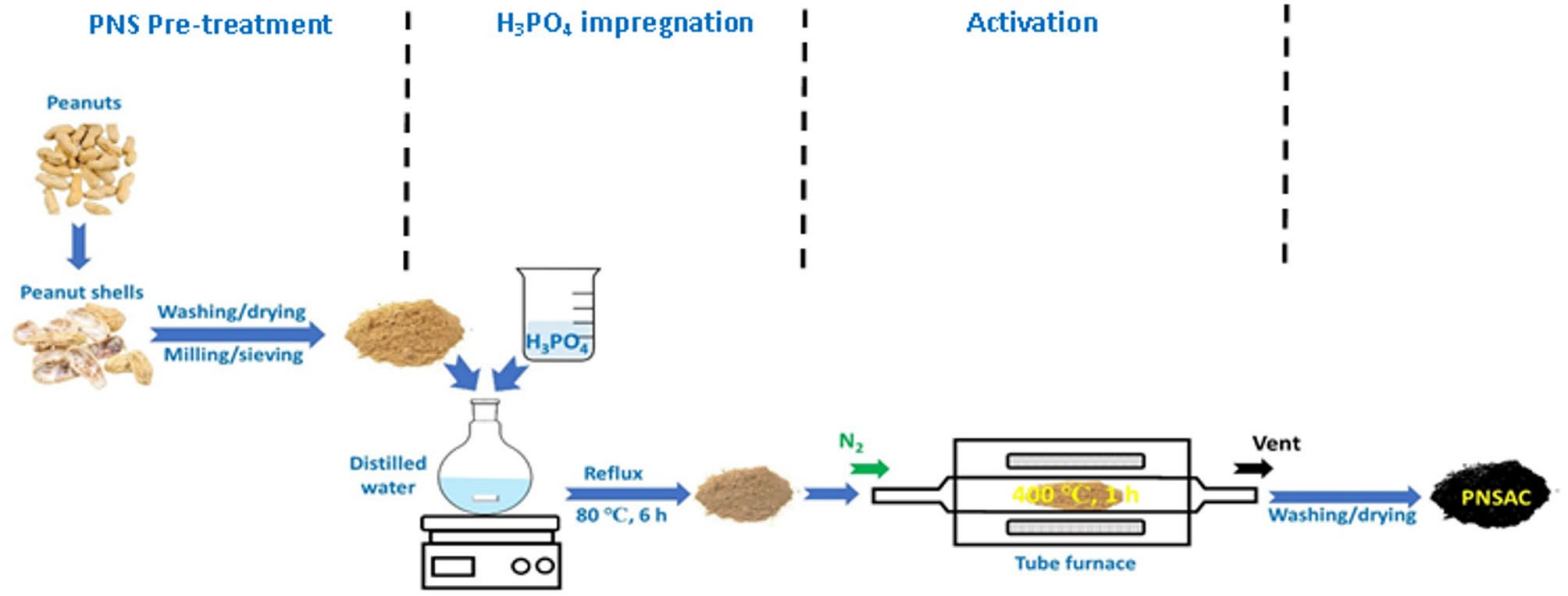
Synthesis process of PNSAC-x from peanut shells (PNS).

The procedure for preparing peanut shell-derived activated carbons is depicted in Fig. 2. Briefly, 5 g of PNS was placed in a conical flask holding 40 mL of distilled water. Concentrated H_3_PO_4_ (85%, w/w) was then introduced to achieve PNS: H_3_PO_4_ weight ratios of 1:1, 1:2, and 1:3.

The mixtures were refluxed with constant stirring at 80 °C for 6 h before being placed in a drying oven at 120 °C until completely dry^42^. The dried samples were then carbonized in a tube furnace at 400 °C for 1 h under an N_2_ flow of 50 mL min^-1^, with a heating ramp of 5 °C min^-1^. The obtained black sample was washed with distilled water until a neutral pH was achieved and then oven-dried to get the final activated carbon samples. The AC samples were designated as PNSAC-1, PNSAC-2, and PNSAC-3, corresponding to PNS: H_3_PO_4_ weight ratios of 1:1, 1:2, and 1:3, respectively. An untreated peanut shell biochar sample was also prepared under identical conditions through direct carbonization of the raw peanut shells at 400 °C for 1 h under N_2_ flow, without the addition of phosphoric acid, and was used as a control.

### Characterization

The PNS precursor and the resulting PNSAC-3 underwent proximate and ultimate analyses. The proximate analysis included calculating moisture, volatile matter, and ash content, following ASTM D2867, ASTM D5832-98, and ASTM D2866-94 standards. The fixed carbon content was then calculated by subtracting the combined percentages of moisture, volatile matter, and ash from 100%^43^. The ultimate analysis involved determining carbon (C), hydrogen (H), nitrogen (N), and sulfur (S) content using an Elemental Vario Macro cube elemental analyzer, with the oxygen (O) content calculated by difference. XRD patterns of the prepared samples were recorded on a Bruker AXS D8 diffractometer employing CuKα radiation (λ = 1.5406 Å), and data were collected by scanning across a 2θ range from 4° to 70°. The textural properties of PNSAC were evaluated through N_2_ adsorption–desorption isotherms at 77 K using a BELSORP-Mini analyzer (BEL Japan, Inc.). The Brunauer–Emmett–Teller (BET) method was applied to determine the specific surface area, while the total pore volume was estimated from the nitrogen uptake at a relative pressure (P/Po) of 0.95. The pore size distribution was further analyzed using the BJH method based on the desorption branch of the isotherm. The FTIR spectra of the samples were obtained using an ATI Unicam (Mattson 936) bench-top spectrophotometer with the KBr pellet technique; the analysis was performed within the wavenumber range of 400–4000 cm^-1^. The surface morphology and structural characteristics of the sample were analyzed using field-emission scanning electron microscopy (FE-SEM, Thermo Scientific Quattro S). High-resolution transmission electron microscopy (HRTEM, JEOL JEM-2100 F) was used to analyze the structural features of the samples. The instrument, operating at 200 kV, provides atomic-scale resolution. This allowed direct observation of lattice fringes and nanoparticle morphology. Raman spectroscopy was performed at room temperature using a SENTERRA Dispersive Raman Microscope. The point of zero charge (PZC) of the adsorbent was determined using the solid addition method, following the procedure described in reference^14^. The zeta potential of PNSAC-3 was determined by dynamic light scattering (DLS) using a Zetasizer Nano ZS (Malvern Panalytical Ltd., Malvern, UK) at 25 °C. The sample was dispersed in deionized water at natural pH (5.6) prior to measurement.

### Batch adsorption experiments

Batch adsorption experiments were accomplished to evaluate the efficiency of PNSAC-x in removing crystal violet (CV) from aqueous solutions. In a typical experiment, a known amount of PNSAC-x was added to a flask containing 20 mg L^-1^ aqueous CV solution. The flask was sealed and shaken at 250 rpm in a water bath kept at room temperature for various durations, covering a range from 2.5 to 30 min. After the adsorption period, the residual CV content was measured using UV-Vis spectroscopy at 584 nm, following the separation of the solid adsorbents through centrifugation. Before conducting the sorption experiments, the adsorbent was vacuum-heated at 150 °C for 12 h to get rid of any moisture.

To reckon the impacts of solution pH, adsorption time, adsorbent dosage, and initial concentrations of CV on adsorption performance, a series of experiments was conducted analogously to the procedure described above. Herein, the pH of the sorption solution was adjusted to the desired level using 0.1 M HCl or 0.1 M NaOH. All adsorption experiments were executed in triplicate to ensure precision and reproducibility.

The CV removal efficiency (%), the amount of CV adsorbed per gram of adsorbent at time t (*q*_t_, mg g^-1^ and at equilibrium (*q*_e_, mg g^-1^ were computed using the following Eqs. (1–3):

1 $$\:Removal\:efficiency=\frac{\left({C}_{o}-{C}_{e}\right)}{{C}_{o}}\times\:100$$

2 $$\:{q}_{t}=\frac{\left({C}_{o}-{C}_{t}\right)V}{m}$$

3 $$\:{q}_{e}=\frac{\left({C}_{o}-{C}_{e}\right)V}{m}$$

Where C_o_ (mg L^− 1^) and C_e_ (mg L^− 1^) are the initial and equilibrium dye concentrations in the solution, respectively, C_t_ (mg L^− 1^) is the dye concentration at time t, V (L) is the volume of CV solution, and (g) is the weight of PNSAC-x used.

### Regeneration and reusability of the adsorbent

Following each adsorption experiment, the mixture was centrifuged, and the spent adsorbent was collected. The used adsorbent was then subjected to a regeneration process using acetic acid as a desorbing agent (100 mL per cycle, repeated three times) under constant stirring at 25 °C to effectively remove the adsorbed crystal violet (CV) molecules from its surface. After regeneration, the adsorbent was thoroughly washed several times with distilled water until a neutral pH was reached, ensuring the complete removal of residual acetic acid. The regenerated material was subsequently vacuum-dried at 70 °C for 12 h prior to reuse. To evaluate the reusability performance, five successive adsorption-desorption cycles were conducted under identical experimental conditions.

## Results and discussion

### Adsorbent screening

**Fig. 3:**
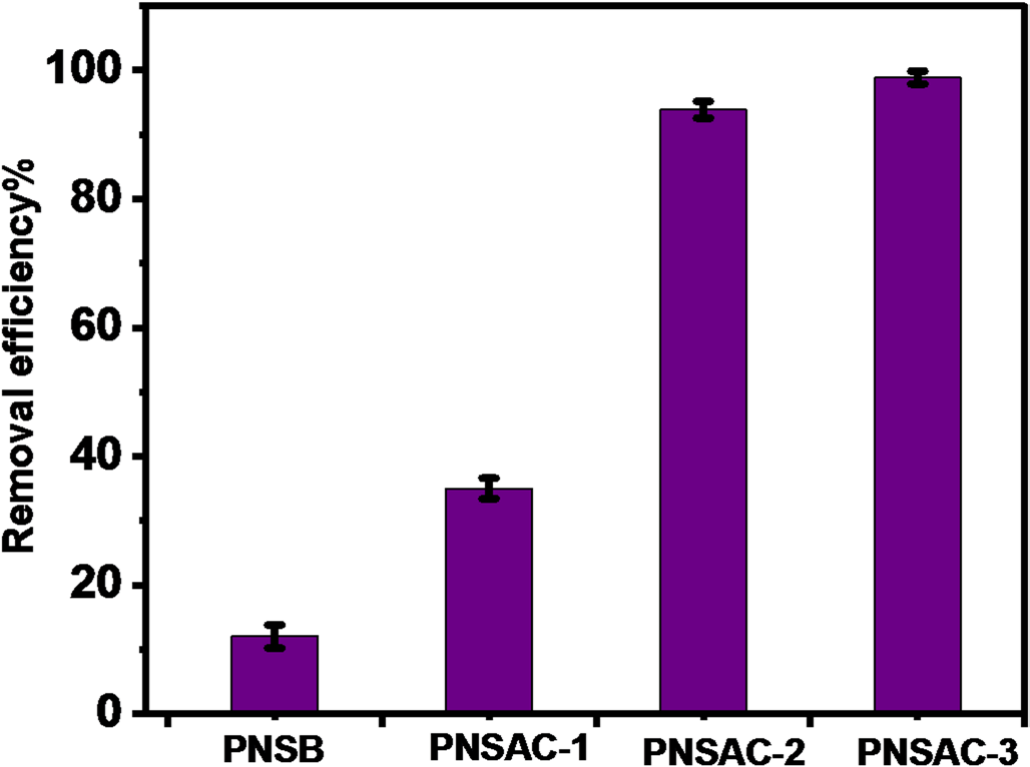
Adsorption efficiency of crystal violet (CV) using non-activated biochar (PNSB) and different PNSAC samples (initial concentration = 20 mg L^−^¹, adsorbent dose = 0.3 g L^−^¹, contact time = 20 min, pH = 5.6, temperature = 25 °C). Error bars represent standard deviation (*n* = 3).

The three activated carbon samples, obtained using different H_3_PO_4_/PNS weight ratios, were examined for their ability to remove CV dye from aqueous solutions. In addition, a biochar sample prepared without chemical activation was also evaluated for comparison purposes. The adsorption experiments were conducted under the following conditions: a contact time of 20 min, ambient temperature, unadjusted pH, and an adsorbent dosage of 0.3 g L^− 1^, with results presented in Fig. 3.

As shown in this figure, the amount of activator plays a critical role in enhancing adsorption performance. The non-activated biochar (PNSB) exhibited a very low dye removal efficiency of only about 12%, confirming the limited adsorption capability in the absence of chemical activation. Similarly, PNSAC-1, obtained using the lowest amount of activator, achieved the lowest removal efficiency of 35%, indicating that the activator amount was insufficient to develop adequate surface functionalization and a sufficient number of active binding sites for dye adsorption. In contrast, PNSAC-2 showed a significant improvement in removal efficiency, reaching 93.88%, which further increased to 98.9% for PNSAC-3. Given its highest removal efficiency, PNSAC-3 was selected for further experiments and characterization.

### Adsorbent characterization

The yield of activated carbon from a biomass precursor, defined as the ratio of the mass of the produced activated carbon to the initial mass of the precursor, is crucial for evaluating both the economic and environmental efficiency of the conversion process. For PNSAC, the yield was approximately 55 wt%, indicating that 1 ton of PNS yields nearly 500 kg of PNSAC under the specified process conditions (PNS: H_3_PO_4_ weight ratio of 1:3 at 400 °C for 1 h). This high yield means adequate utilization of the biomass precursor and positively impacts both the financial viability and environmental sustainability of activated carbon production from PNS.

Table 1 recaps the ultimate analysis results of the raw peanut shells (PNS) and their activated carbon derivative (PNSAC-3). Ultimate analysis reveals significant compositional changes after carbonization. The raw shells consist of oxygen (47.8%), carbon (45.19%), nitrogen (1.43%), sulfur (0.4%), and hydrogen (5.46%). After carbonization, carbon content rises to 65%, accompanied by a sharp lowering in hydrogen to 1.25%, nitrogen to 1.04%, with sulfur being completely removed. Oxygen content also decreases substantially to 32.96%. These changes reflect the loss of volatiles and enhanced carbon purity^13^. The H/C atomic ratio drops from 1.45 in peanut shells to 0.23 in derived carbon, while the O/C ratio declines from 0.78 to 0.38, reflecting the increased aromaticity and thermal stability of the carbon material, which enhances its suitability for advanced applications such as energy storage and adsorption processes^44^.

**Table 1:** Product yield and ultimate analysis of the prepared activated carbon (PNSAC-3).

Parameter	PNS	PNSAC-3
**Product yield (%)**	N/A	55.20
**Ultimate analysis**		
C (%)	45.19	65.00
H (%)	5.46	1.25
N (%)	1.43	1.04
S (%)	0.41	0.00
O (%) (by difference)	47.80	32.96
H/C	1.45	0.23
O/C	0.78	0.38

**Fig. 4:**
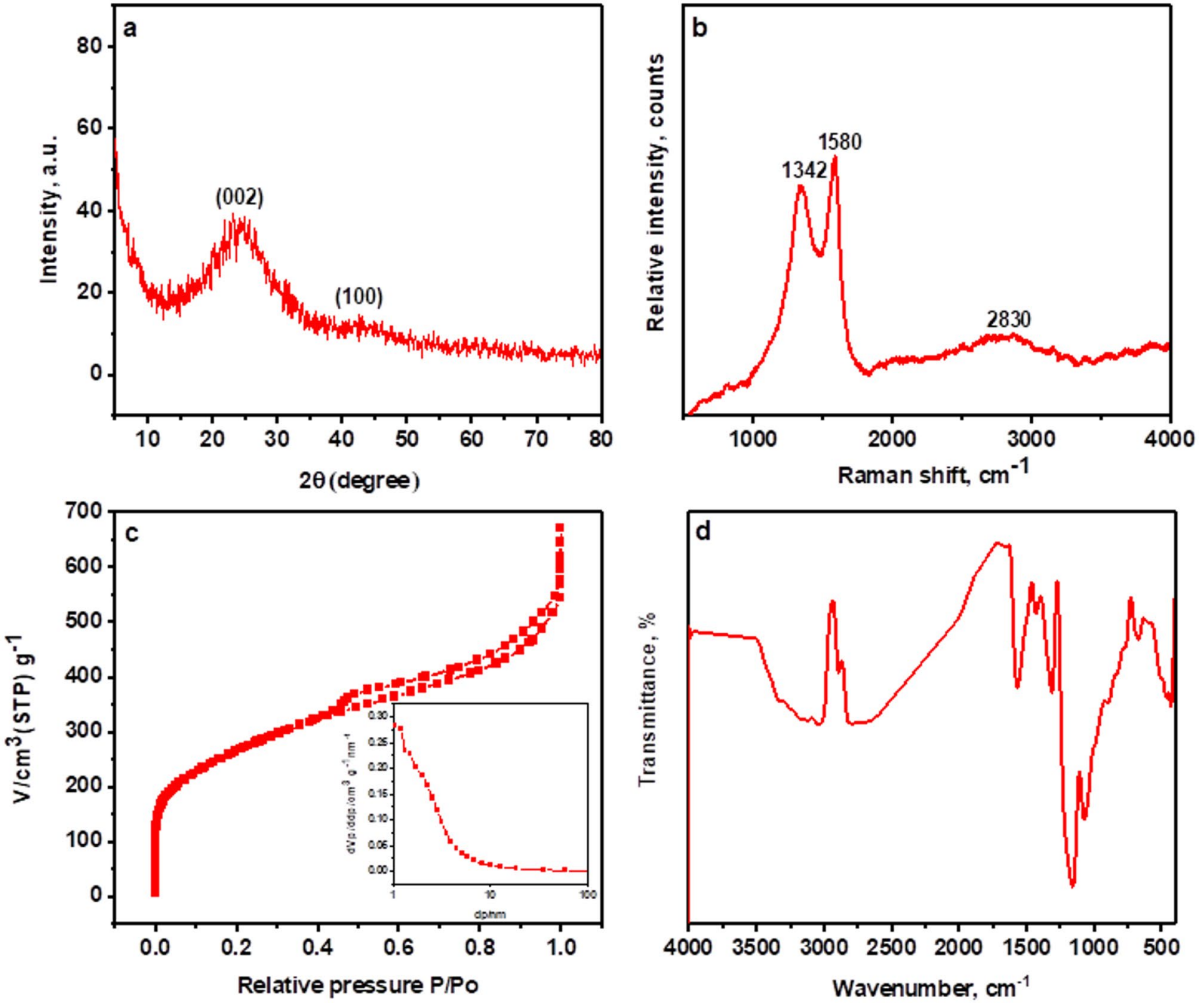
(**a**) XRD pattern, (**b**) Raman spectrum, (**c**) N_2_ adsorption-desorption isotherm with BJH pore size distribution inset, and (**d**) FTIR spectrum of the PNSAC-3 sample.

The X-ray diffractogram of the PNSAC-3 sample, depicted in Fig. 4a, displays two wide diffraction peaks at approximately 23° and 43°, associated with the (002) and (100) reflections of carbons, respectively (JCPDS 75–1621)^20,45^. The broadness and low intensity of these peaks indicate that the sample possesses an amorphous structure with a low level of graphitization^13,44^. The XRD pattern of the raw PNS precursor is provided in Supplementary Information (Fig. S1) for structural comparison. The characteristic cellulose I reflections at 2θ ≈ 22° and ~ 17° are no longer discernible in PNSAC-3, replaced by broad graphitic humps at ~ 23° and ~ 43°^46,47^, confirming the complete decomposition of the lignocellulosic framework and the successful formation of a disordered amorphous carbon structure upon H_3_PO_4_ chemical activation^48^. This is consistent with the ~ 100-fold enhancement in BET surface area observed for PNSAC-3 compared to the untreated biochar.

Raman spectra of the PNSAC sample (Fig. 4b) display two distinct bands at 1342 and 1580 cm^− 1^, corresponding to the D and G bands, respectively^13^. The G band, originating from in-plane vibrations of the *sp*^2^ carbon bonds, reflects the presence of graphitic ordering, while the D band, linked to out-of-plane vibrations, is associated with structural defects and disorder in the carbon matrix^14,49^. The intensity ratio of these bands (I_D_/I_G_) is calculated to be 0.86, suggesting a significant level of structural disorder, which is consistent with the broad diffraction peaks observed in the XRD pattern (Fig. 4a). This structural disorder is typical for biomass-derived activated carbons and contributes to increased porosity and surface area, enhancing their performance for adsorption purposes^13^. Additionally, a weak 2D peak is observed at 2830 cm^[− 150^.

To assess the pore characteristics of the synthesized adsorbent, N_2_ physisorption measurement was executed at 77 K, and the resulting N_2_ adsorption isotherm is shown in Fig. 4c. PNSAC-3 exhibits a Type IV isotherm, indicative of a mesoporous structure (pore diameters between 2 and 50 nm). An obvious H3 hysteresis loop appears at P/Po above 0.4, without a clear saturation plateau at high relative pressures, indicating capillary condensation in mesopores. This hysteresis is associated with slit-shaped mesopores with non-uniform sizes^51^. The pore size distribution determined using the BJH model (inset in Fig. 4c) further corroborates the mesoporous nature suggested by the isotherm Type and the H3 hysteresis loop, with the majority of pores centered around 2–4 nm. The BET-specific surface area (*S*_BET_) of the PNSAC-3 sample was determined to be 934.8 m^2^ g^− 1^, with a pore volume of 0.823 cm^3^ g^− 1^. These enhanced texture properties, in terms of large *S*_BET_ and mesoporous structure, ensure an extensive surface for adsorbate molecules to interact with and provide easy access to the adsorption sites, thereby enhancing overall adsorption capacity and efficiency. Notably, such favorable characteristics were achieved at a relatively low carbonization temperature of 400 °C, which improves the material’s economic viability, environmental friendliness, and sustainability for water remediation applications. For comparison, the textural properties of the untreated biochar (PNSB ) were also evaluated (Fig. S2). The untreated biochar exhibited a BET surface area of only 9.4 m² g^− 1^ and a pore volume of 0.098 cm^3^ g^− 1^. This confirms that thermal treatment alone at 400 °C is insufficient to generate a well-developed porous structure in the absence of a chemical activating agent, demonstrating that H_3_PO_4_ activation is the key factor responsible for the ~ 100-fold enhancement in surface area in PNSAC-3.

The FTIR spectrum of raw PNS (Fig. S3a) exhibits characteristic absorption bands of lignocellulosic biomass. The broad absorption band at 3341 cm^-^¹ is attributed to O-H stretching vibrations of hydroxyl groups in cellulose, hemicellulose, and adsorbed moisture^52–54^. The band at 2926 cm^-^¹ corresponds to C-H stretching vibrations of aliphatic –CH_2_ and –CH_3_ groups in hemicellulose and lignin^52,54,55^. The peak at 1738 cm^-^¹ is assigned to the C = O stretching of ester linkages in hemicellulose, confirming the intact polysaccharide structure^53–55^. The bands at 1648 and 1513 cm^-^¹ are attributed to C = C aromatic stretching vibrations and skeletal vibrations of the aromatic rings in lignin, respectively^52,54,55^. The absorptions at 1425, 1320, and 1262 cm^-^¹ correspond to C-H bending, C-O-H bending in phenolic groups, and C-O stretching in ether and ester linkages of lignin, respectively^52,55^.

In comparison, the FTIR spectrum of the untreated biochar (Fig. S3b) shows a marked simplification of the spectral features following pyrolysis at 400 °C without chemical activation. The disappearance of the 1738 cm^-^¹ band confirms the thermal decomposition of hemicellulose ester linkages during carbonization^56^. Similarly, the absence of the 2926 cm^-^¹ band indicates the elimination of aliphatic C-H groups^56,57^ The residual broad O-H band at 3397 cm^-^¹ suggests the partial retention of hydroxyl functionalities^56–58^, while the bands at 1411 and 1020 cm^-^¹ indicate the presence of residual aromatic C = C and C-O stretching vibrations, respectively, reflecting a partially carbonized structure^57^. These changes confirm partial devolatilization, aromatization, and structural condensation during pyrolysis.

Upon H_3_PO_4_ chemical activation at 400 °C, the PNSAC-3 spectrum (Fig. 4d) exhibits a distinctly different profile, with significant structural modification. The strong absorption bands observed within the 1000–1200 cm^-^¹ region are attributed to C-O stretching vibrations as well as phosphorus-related functionalities, such as P–O–C and P = O groups formed during activation^42,59,60^. The appearance and intensification of these bands confirm the successful chemical modification of the carbon framework. Furthermore, the disappearance or weakening of biomass-related peaks demonstrates the extensive decomposition of the original lignocellulosic structure and formation of a disordered porous carbon network. These oxygenated and phosphorus-containing functional groups are expected to enhance the adsorption performance of PNSAC-3 through hydrogen bonding, electrostatic interactions, and surface interaction mechanisms.

**Fig. 5:**
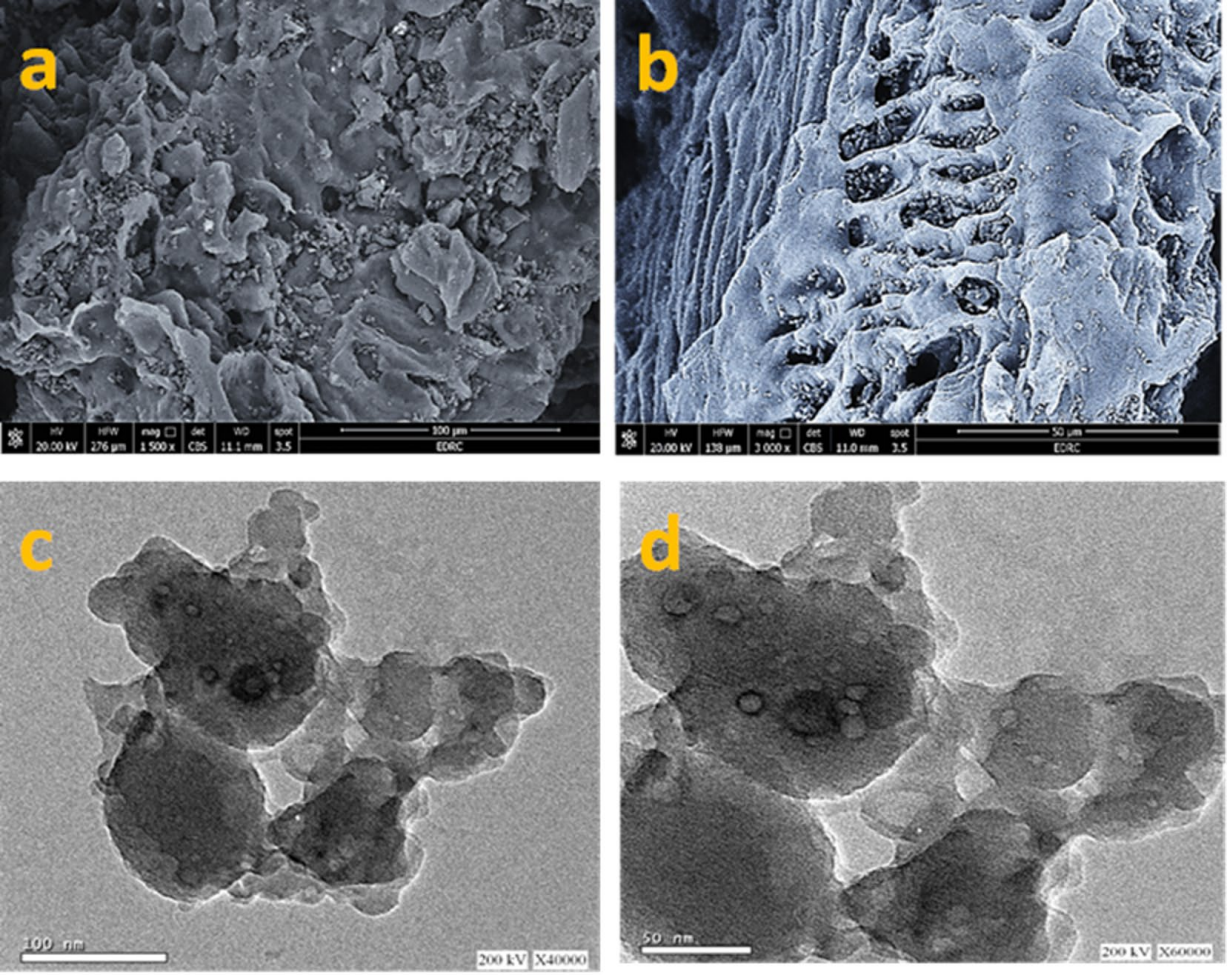
(**a**,** b**) SEM micrographs at different magnifications, (**c**,** d**) HR-TEM images of the PNSAC-3 sample.

The morphology of the PNSAC-3 sample was thoroughly scrutinized using SEM and HR-TEM, as shown in Fig. 5a-d. The SEM micrographs clearly reveal a highly rough and irregular surface morphology with visible pore formation and cavities. This highly porous structure is attributed to H_3_PO_4_ chemical activation, which facilitates the release of moisture and volatile materials, creating porous spaces. Such a structure enhances the accessibility of the adsorption sites and improves mass transfer efficiency, which is beneficial for adsorption. Moreover, HR-TEM images confirm the amorphous and porous nature of the carbon material, consistent with the Raman spectroscopy and N_2_ physisorption results. This amorphous structure can also contribute positively to adsorption performance.

The SEM image of the raw PNS precursor (Fig. S4) reveals a smooth, non-porous layered morphology, in stark contrast to the rough and porous surface observed for PNSAC-3. This confirms that H_3_PO_4_ activation is the primary driver for the observed morphological and textural transformation.

### Cost estimation of prepared activated carbon

A detailed cost analysis of activated carbon (AC) adsorbents is essential for evaluating their economic feasibility and potential real-world application in water treatment processes. The overall production cost covers various stages, including waste collection, pre-treatment (e.g., washing, size reduction), pyrolysis, chemical activation, and neutralization^45^. Key factors, such as waste availability, preparation conditions, energy requirements, and adsorbent lifecycle, have a paramount influence on the overall cost of AC production^45^.

In this context, producing activated carbons from agricultural waste, such as peanut shells (PNS), offers notable advantages. This approach not only transforms low-cost, abundant agricultural residues but also yields activated carbon with a high surface area and pore volume, which is highly effective for removing water pollutants like crystal violet (CV).

The cost estimate is based on an overall yield of approximately 55%. High product yield reduces the reactor volume, chemical consumption, and energy use, ultimately lower production costs and improving commercial scalability. The estimated cost of producing 1 kg of PNSAC using H_3_PO_4_ as an activating agent is detailed in Table 2, according to the procedure described in Sect.  2.2. Note that the cost of raw peanut shells is not included in the estimate, as they were obtained free of charge.

As shown in Table 2, the estimated production cost is strongly dependent on the grade of phosphoric acid used. When analytical-grade phosphoric acid is used, the total production cost is approximately 780 LE kg^-1^, whereas with commercial-grade phosphoric acid, it significantly drops to about 115 LE kg^-1^. This difference highlights the importance of selecting cost-effective reagents for scalable production. Compared to commercially available activated carbon, typically priced at 150–250 LE kg^-1^, PNSAC-3 presents a competitive and cost-effective alternative. Based on the data in Table 2, the chemical activator emerges as the primary cost driver for PNSAC production. Consequently, implementing acid recovery or recycling strategies would not only minimize the ecological footprint by preventing phosphate-related environmental issues but also significantly enhance the economic feasibility and commercial scalability of the process^61^. In addition, the prepared adsorbent exhibited excellent regeneration performance, maintaining more than 94% removal efficiency after five consecutive cycles, thereby extending its operational lifetime and reducing replacement frequency.

**Table 2:** The estimated cost per kilogram of PNSAC production from PNS (LE kg^− 1^).

Item	Cost, LE kg^− 1^	Cost, LE kg^− 1^
Raw material (Peanut shells)	Supplied free of cost	Supplied free of cost
Phosphoric acid (H_3_PO_4_)	740	75
Distilled water	water is from internal laboratory setup	water is from internal laboratory setup
Total electricity cost (Size reduction, drying, chemical activation, and carbonization)	40	40
Net cost	780	115

To provide a standardized economic assessment, the adsorption cost per gram of adsorbate (AC) was estimated according to the methodology reported in recent literature (Eq. 4)^62^. The adsorption cost was calculated based on the total preparation cost of the adsorbent, including chemical and energy expenses, divided by the maximum adsorption capacity. The production cost using commercial-grade H_3_PO_4_ was approximately 0.0024 USD g^− 1^ adsorbent, while the maximum adsorption capacity toward crystal violet reached 145.35 mg g^− 1^. Accordingly, the estimated adsorption cost was 0.0165 USD g^− 1^ of removed CV. According to previously established benchmarks, adsorbents with a cost lower than 1 USD g^− 1^ are considered highly economical. It should be noted that the present cost estimation represents a preliminary laboratory-scale economic assessment primarily focused on raw material and energy consumption. Additional factors such as labor cost, equipment depreciation, facility overhead, solvent recovery, and waste management were not included and should be considered in future large-scale assessments.

4 $$\:{C}_{ADS}=\:\frac{{\mathrm{C}}_{\mathrm{c}\mathrm{h}\mathrm{e}\mathrm{m}}+\:{\mathrm{C}}_{\mathrm{e}\mathrm{n}\mathrm{e}\mathrm{r}\mathrm{g}\mathrm{y}}}{{\mathrm{q}}_{\mathrm{m}\mathrm{a}\mathrm{x}}\:\:\times\:\:{10}^{-3}}$$

Where the C_ADS_ (USD g^− 1^) is the adsorption cost per gram of adsorbate, C_chem_ (USD g^− 1^) is the chemical cost per gram of adsorbent, C_energy_ (USD g^− 1^) is the energy cost per gram of adsorbent, and q (mg g^− 1^) is the maximum adsorption capacity.

### Batch adsorption experiments

#### Effect of adsorbent dose on CV adsorption

**Fig. 6:**
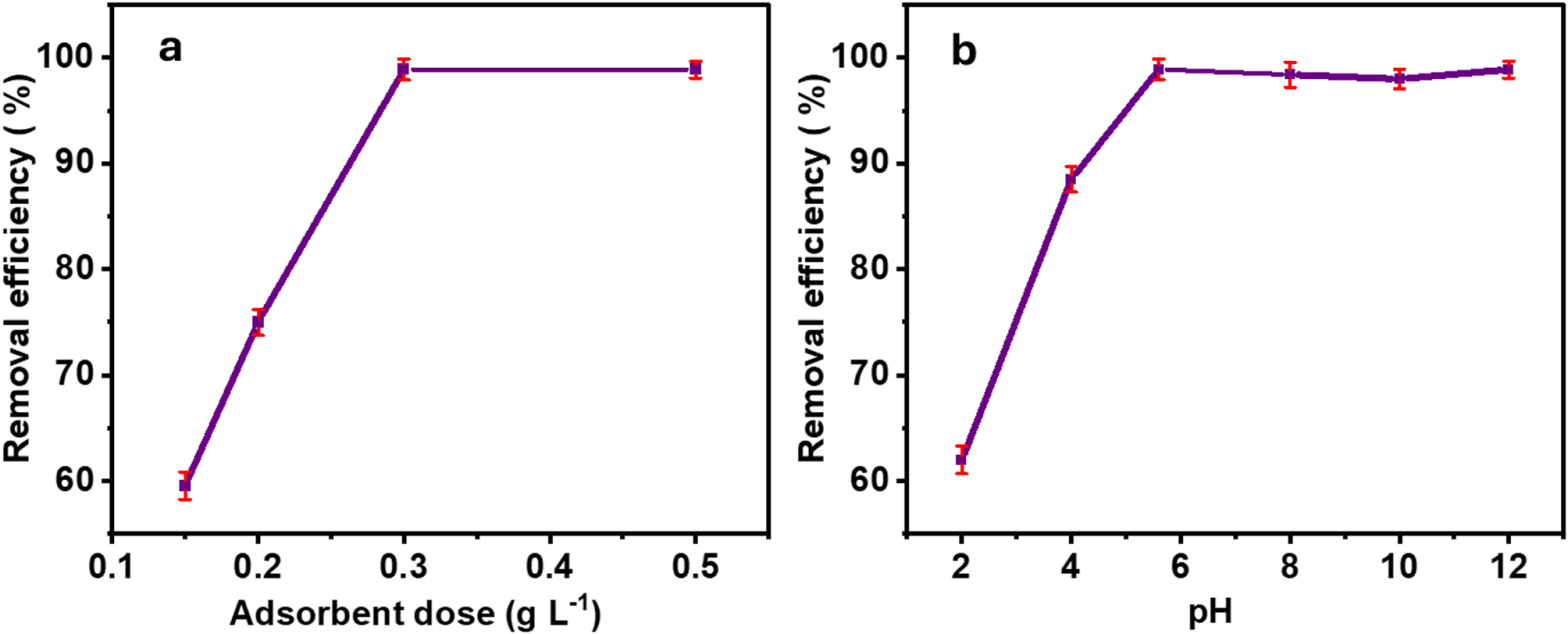
**(a)** Effect of adsorbent dose on the removal efficiency of CV by PNSAC-3 (initial concentration = 20 mg L^− 1^, contact time = 20 min, pH = 5.6, temperature = 25 °C).(**b**) Effect of pH on the removal efficiency of CV by PNSAC-3 (initial concentration = 20 mg L^− 1^, contact time = 20 min, adsorbent dose = 0.3 g L^− 1^, temperature = 25 °C). Error bars represent standard deviation (*n* = 3).

To determine the optimal dose of the PNSAC-3 adsorbent for effective adsorption, a series of tests was executed in which the volume of the CV solution (20 mg L^− 1^) was fixed at 10 ml, and various weights of PNSAC-3 were introduced to achieve PNSAC-3 doses ranging from 0.15 to 0.5 g L^− 1^. These experiments were performed for 20 min at natural pH and a temperature of 25 °C. The results, manifested in Fig. 6a, revealed that the adsorption efficiency increased with the PNSAC-3 dose from 0.15 g L^− 1^ to 0.3 g L^− 1^. Beyond 0.3 g L^− 1^, the adsorption efficiency remained almost unchanged. The increased exposed surface areas and the greater number of effective binding sites provided by the adsorbent with the increase in its amount from 0.15 to 0.3 g L^− 1^, rendered the CV removal efficacy rise^13,63,64^. However, the plateau in CV removal percentage at doses above 0.3 g L^− 1^ might be a consequence of the conglomeration of the PNSAC-3 particles, and the associated increase in the diffusion path length for CV species, and reduction in the available surface area and the number of binding centers participating in the sorption process^63,65,66^. Therefore, 0.3 g L^− 1^ was selected as the optimum adsorbent dose, as it provided the highest effective adsorption performance while avoiding the unnecessary use of additional adsorbent in all subsequent experiments.

#### Effect of pH on CV adsorption and the proposed adsorption mechanism

Initial pH is a decisive parameter affecting the uptake of organic dyes from aqueous media, as it governs both the surface charge of the adsorbent and the ionization state of the dye, thereby controlling the interaction between them^65,67^. The effect of pH on CV adsorption was investigated over a range of 2–12, while keeping the initial CV concentration (20 mg L^− 1^), contact time (20 min), and adsorbent dosage (0.3 g L^− 1^) constant. The results shown in Fig. 6b indicate that CV removal efficiency increases with rising pH, peaking at the natural pH of the solution (5.6), and remaining nearly constant at higher pH levels.

Crystal violet (CV) is predominantly cationic across the studied pH range due to its low pKa value (0.8)^68^. The point of zero charge (PZC) of PNSAC-3 was found to be 3.8. Thus, the adsorbent surface is neutral at pH 3.8, positively charged below this value, and negatively charged above it. To further support this, zeta potential measurements were conducted at the natural pH of the solution (pH 5.6), yielding a value of **−** 19 mV (Fig. S5). This negative potential confirms that at pH > PZC, the surface functional groups undergo deprotonation, rendering the surface negatively charged. Conversely, at pH < PZC, the surface becomes protonated.

At a pH of 2 (below the PZC), the PNSAC-3 surface is positively charged, which induces electrostatic repulsion with the cationic crystal violet (CV) species, leading to a decrease in adsorption efficiency compared to neutral and alkaline conditions. However, despite the presence of electrostatic repulsive forces, a moderate adsorption level (~ 60%) is still observed. This behavior suggests that the adsorption process is not solely governed by electrostatic interactions. Instead, non-electrostatic mechanisms play An important role under highly acidic conditions. These include π–π interactions between the aromatic rings of CV and the graphitic domains of PNSAC-3, hydrogen bonding between nitrogen-containing functional groups in CV and surface oxygen-containing groups, as well as hydrophobic interactions that facilitate the accumulation of dye molecules on the carbon surface^65,69,70^. In addition, the highly porous structure of PNSAC-3 enables pore-filling effects, which further enhance dye uptake even under unfavorable electrostatic conditions^69,70^.

**Fig. 7:**
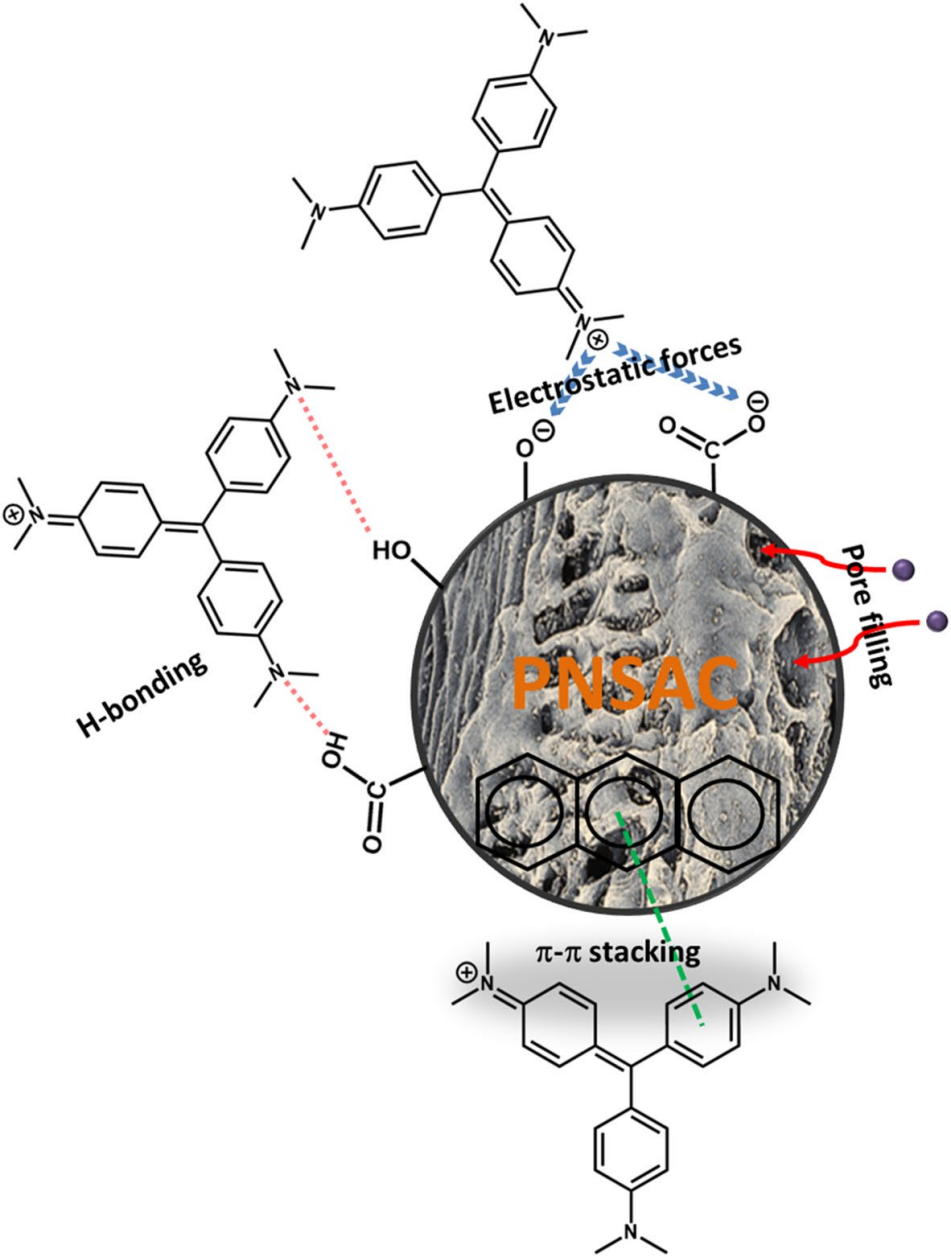
Proposed adsorption mechanism between PNSAC-3 and the predominant cationic form of crystal violet under the optimum adsorption conditions (natural pH = 5.6).

As the pH increases beyond the PZC, the surface becomes negatively charged, which enhances electrostatic attraction with cationic CV molecules, leading to high and nearly constant removal efficiency^65,67,69,70^. Based on BET and SEM analyses confirming the highly porous structure of PNSAC-3, together with the ~ 1.4 nm size of CV, pore filling likely contributes to adsorption across the entire pH range^69,70^. The proposed adsorption mechanism of CV onto PNSAC-3 is illustrated in Fig. 7. Based on these results, pH 5.6 (natural pH) was selected as the optimum condition for subsequent experiments due to its high removal efficiency without the need for pH adjustment.

#### Equilibrium modelling

**Fig. 8:**
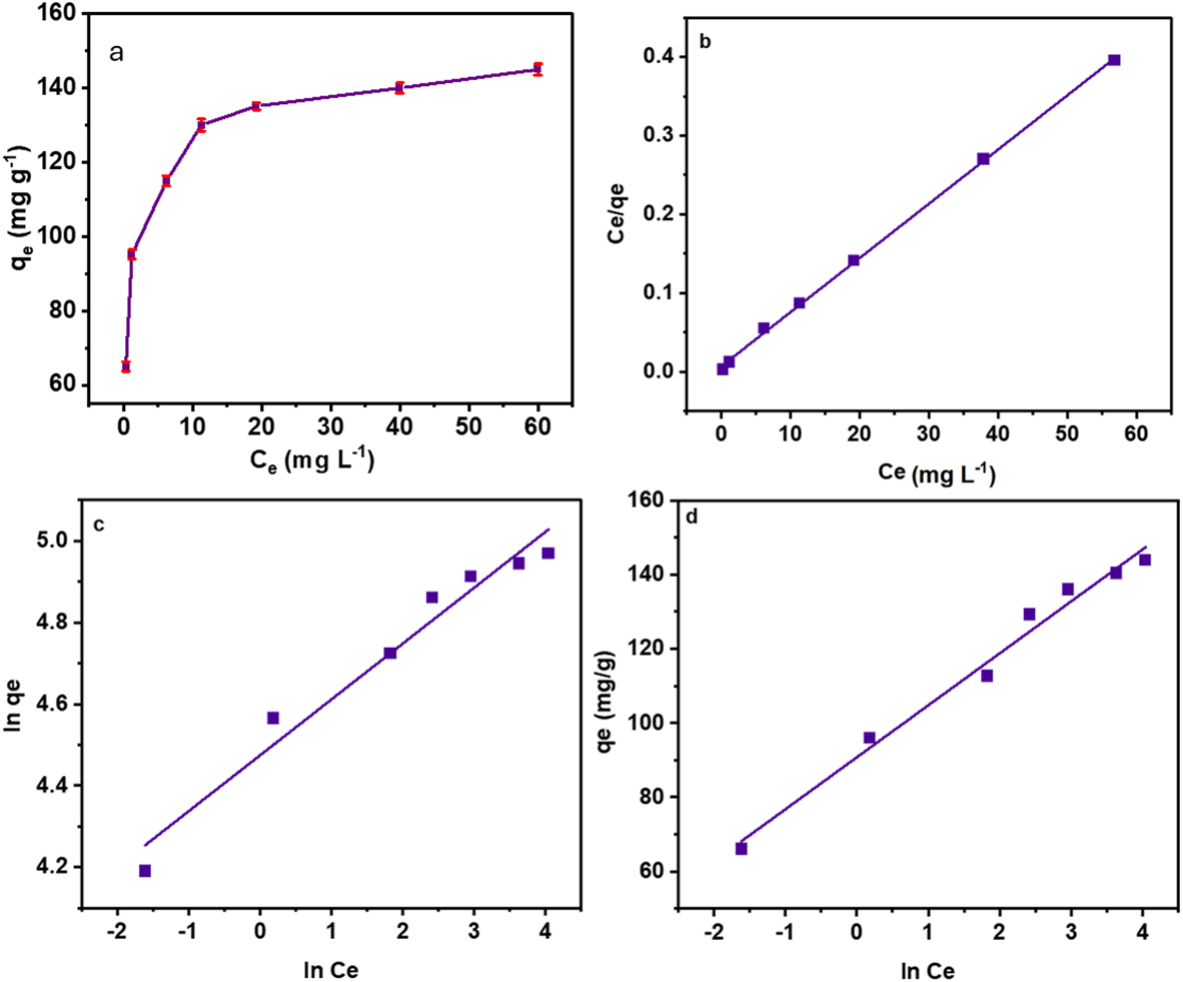
(**a**) Equilibrium adsorption isotherm of CV onto PNSAC-3 at 25 °C (adsorbent dose = 0.3 g L⁻¹, pH = 5.6, contact time = 20 min); error bars represent standard deviation (*n* = 3). Linear fitting plots of (**b**) Langmuir, (**c**) Freundlich, and (**d**)Temkin isotherm models.

The adsorption isotherm analysis provides valuable insights into the mechanism of CV adsorption on PNSAC-3. As shown in Fig. 8a, the plot of q_e_ against C_e_ reveals a steep initial rise in the quantity of CV adsorbed at low CV concentrations, indicating a strong affinity between CV molecules and PNSAC-3 due to the abundant available binding sites. This is followed by a more gradual increase, as the sites become increasingly occupied, ultimately reaching a plateau, indicating equilibrium.

Three isotherm models, Langmuir (Eq. 5), Freundlich (Eq. 6), and Temkin (Eq. 7) isotherms, were employed to analyze the experimental data. The corresponding linearized plots are manifested in Fig. 8b-d, and the pertinent parameters with regression coefficients are recapitulated in Table 3.

5 $$\:\frac{{C}_{e}}{{Q}_{e}}=\frac{1}{{q}_{max}{K}_{L}}+\frac{{C}_{e}}{{q}_{max}}$$

6 $$\:\:{\:\:\:\:ln}{q}_{e}={ln}{K}_{F}+\frac{1}{n}{ln}{C}_{e}$$

7 $$\:{q}_{e}=B\mathrm{ln}A+B\mathrm{ln}{C}_{e}$$

where *q*_*max*_*(mg g*^-1^*)* is the maximum adsorption capacity; *K*_*L*_*(L mg*^-1^*)* is the Langmuir adsorption constant; *K*_*F*_*(mg g*^-1^*)(L mg*^-1^*)*^*1/n*^*)* is the Freundlich constant related to adsorption capacity; n is a dimensionless constant indicating the favorability of the adsorption process, A is the Temkin isotherm equilibrium binding constant, and B is a constant related to the heat of adsorption.

As summarized in Table 3, the Langmuir model provided the best fit with the highest correlation coefficient (R^2^ **=** 0.999). This indicates a monolayer adsorption mechanism on a surface containing a finite number of identical sites, where adsorption is uniform, and no lateral interaction between the adsorbed molecules occurs^66,71,72^. The calculated maximum adsorption capacity (q_max_) was 145.35 mg g^− 1^, reflecting the high efficiency of PNSAC-3. Langmuir constant (K_L_) is associated with the binding energy and affinity of the adsorbent for the adsorbate. Higher $\:{K}_{L}$values generally indicate stronger adsorbate–adsorbent interactions and greater adsorption affinity. In the present study, the relatively high *K*_*L*_ value indicates a strong attraction between the CV molecules and the PNSAC-3 surface^73^.

The dimensionless equilibrium parameter, R_L_, also known as the separation factor, was employed to characterize the nature of the adsorption process (Eq. 8). The value of R_L_ indicates whether the adsorption is unfavorable (R_L_ > 1), linear (R_L_ = 1), favorable (0 < R_L_ < 1), or irreversible (R_L_ = 0)^64,66^. In this study, the R_L_ values for CV adsorption onto PNSAC-3 over the entire CV concentration range (Table 4) were found to be within the range of (0.05 − 0.01). This confirms that the adsorption process is highly favorable. Furthermore, the observed decrease in R_L_ values with increasing initial CV concentration indicates that the interaction becomes more favorable and shifts toward irreversibility at higher concentrations, highlighting the strong affinity of PNSAC-3 for CV molecules^74^.

8 $$\:{R}_{L}=1/\left(1+{K}_{L}{C}_{o}\right)$$

The Freundlich constant (n) serves as an indicator of adsorption favorability. A value of n greater than unity indicates favorable adsorption behavior^75,76^. In the present study, the calculated n value was 7.3 (which corresponds to 1/*n* = 0.137), confirming that the adsorption of CV onto PNSAC-3 is highly favorable and suggesting a strong affinity between the adsorbent surface and CV molecules.

**Table 3:** Isotherm model Parameters of CV adsorption onto PNSAC-3 at 25 °C.

Isotherm Model	Parameters	PNSAC-3
**Langmuir Isotherm**	*q*_*max*_ *(mg g*^− 1^*)*	145.35
	*K*_*L*_ *(L m g*^− 1^*)*	0.946
	R^2^	0.999
**Freundlich Isotherm**	*K*_*F*_ *(mg g*^− 1^*)(L mg*^− 1^*)* ^*1/n*^	87.80
	*n*	7.30
	*R* ^*2*^	0.957
**Temkin Isotherm**	*A (L g* ^− 1^ *)*	651
	*B (J mol* ^− 1^ *)*	14
	*R* ^*2*^	0.98

**Table 4:** R_L_ values based on Langmuir adsorption at different CV initial concentrations.

C_o_ (mg l^− 1^)	*R*_L_ values
20	0.050
30	0.034
40	0.0257
50	0.0207
60	0.0173
80	0.013
100	0.01

#### Sorption kinetics

**Fig. 9:**
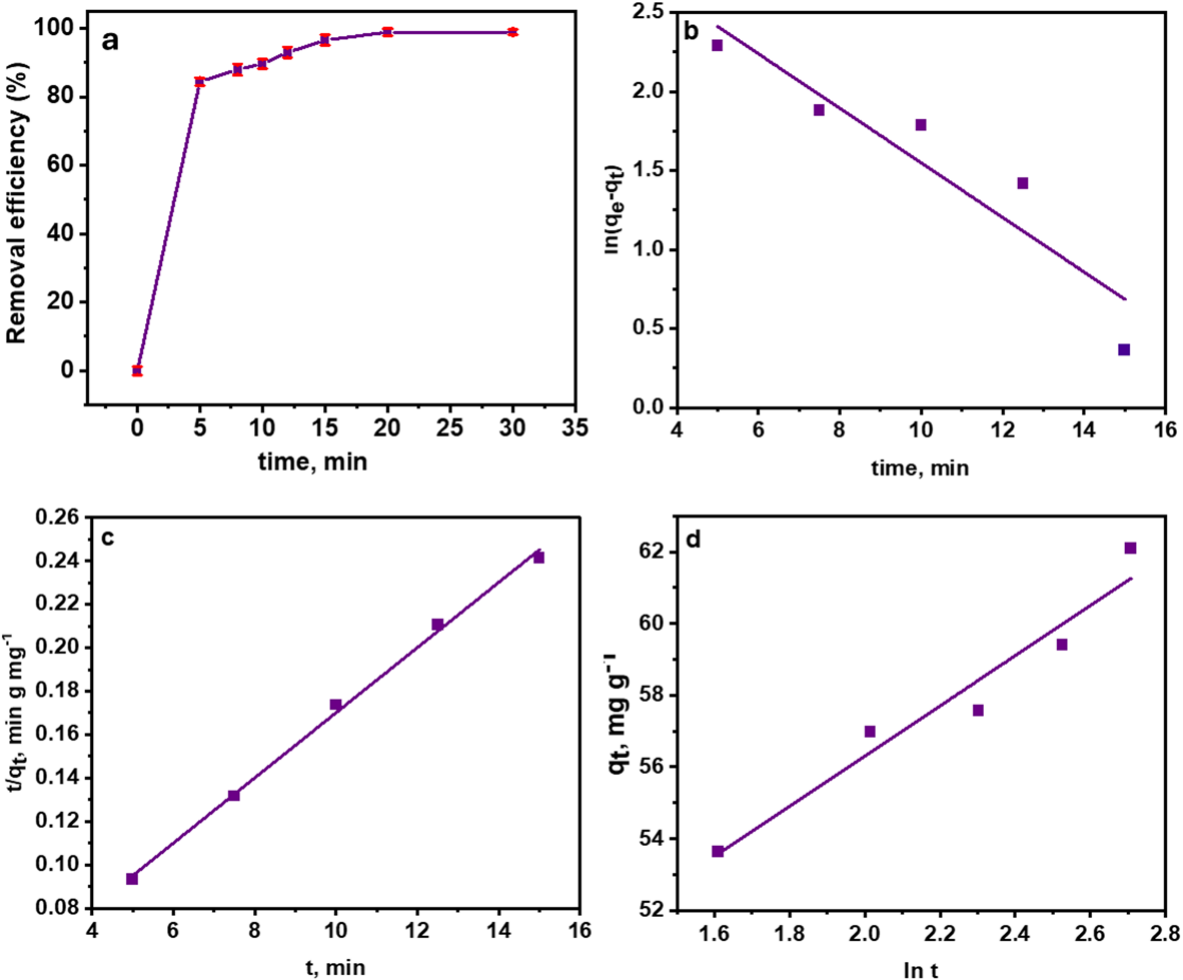
(**a**) Effect of contact time on the removal efficiency of CV by PNSAC-3 (initial concentration = 20 mg L^− 1^, adsorbent dose = 0.3 g L^− 1^, pH = 5.6, temperature = 25 °C); error bars represent standard deviation (*n* = 3). Kinetic model fitting plots: (**b**) pseudo-first-order, (**c**) pseudo-second-order, and (**d**) Elovich models.

Figure 9a displays the variation in CV removal efficiency as a function of contact time. The experiments were conducted at an initial CV concentration of 20 mg L^-1^, an adsorbent dosage of 0.3 g L^-1^, and natural pH. The adsorption process reached equilibrium within 20 min. The entire adsorption process can be divided into three distinct stages: 1–5, 5–20, and 20–30 min. In the initial stage (0–5 min), a rapid increase in adsorption capacity was observed, reaching approximately 53.63 mg g^-1^(about 84.42% of the total capacity, 63.6 mg g^-1^, which is attributed to the ample availability of vacant active binding centers on the PNSAC**-**3 surface^65,77^.

In the second stage, the subsequent 15 min, the uptake rate slowed as the active binding centers became increasingly occupied. Eventually, after about 20 min, the adsorption plateaued, indicating that most of the binding centers were occupied, and adsorption equilibrium had been achieved^63,65^. Accordingly, 20 min was selected as the optimum contact time for further experiments.

The kinetic data were fitted to the pseudo-first order (PFO), pseudo-second-order (PSO), and Elovich models (Eqs. 9–11).

9 $$\:\mathrm{ln}\left({q}_{e}-{q}_{t}\right)=\mathrm{ln}{q}_{e}-{k}_{1}t$$

10 $$\:\:\:\:\:\:\:\:\:\:\frac{t}{{q}_{t}}=\frac{1}{{k}_{2}{q}_{e}^{2}}+\frac{t}{{q}_{e}}$$

11 $$\:\:\:\:\:\:\:\:\:\:{q}_{t}=\frac{1}{\beta\:}\mathrm{ln}\alpha\:\beta\:+\frac{1}{\beta\:}\:\mathrm{ln}t$$

where *k*_*1*_ (min^− 1^) is the rate constant of the pseudo-first-order model, *k*_*2*_ (g mg^− 1^ min^− 1^) is the rate constant of the pseudo-second-order, α (m g^− 1^.min^− 1^) is the Elovich initial adsorption rate constant, and β (g mg^− 1^) is the Elovich desorption rate constant.

The linearized forms are manifested in Fig. 9b-d, and the kinetic parameters derived from these plots are collected in Table 5. The PSO model, which assumes that the adsorption rate is proportional to the square of the difference between the equilibrium adsorption capacity and the instantaneous uptake^78^, fits the removal process more aptly than the two models, as evidenced by the highest regression coefficient (R^2^). This suitability is further confirmed by the excellent consistency between the experimental adsorption capacity (63.6 mg g^− 1^) and the model-predicted value (66.66 mg g^− 1^). The good agreement with the PSO model suggests that the adsorption process is mainly governed by surface-controlled adsorption kinetics and strong adsorbate-adsorbent interactions. Although the PSO model is frequently associated with chemisorption-related systems^63,69,77,79^. It does not by itself constitute definitive proof of a chemisorption mechanism involving valence forces or electron sharing/exchange between the PNSAC-3 surface and CV molecule.

In addition, the high R^2^ value obtained for the Elovich model suggests the presence of heterogeneous adsorption sites and energetically diverse surface interactions on the adsorbent surface^77,80^. The relatively high value of the initial adsorption rate constant **(**α) reflects the rapid uptake of the CV molecules at the early stage of the process, driven by the abundance of available active sites on the PNSAC-3 surface. Meanwhile, the Elovich desorption rate constant (β) (0.14 g mg^− 1^) reflects a progressive decline in adsorption kinetics as the active sites become increasingly occupied. Notably, the much higher magnitude of α compared to β indicates that the possible release of the adsorbed CV molecules back to the solution phase is relatively limited under the studied conditions.

**Table 5:** Kinetic model parameters of CV adsorption onto PNSAC**-**3.

Kinetic Model	Parameters	PNSAC-3
Pseudo-First-Order	*k*_*1*_ *(L min*^*− 1*^*)*	0.17
	*q*_*e*_ *(mg g*^*− 1*^*)*	26.33
	*R* ^*2*^	0.87
Pseudo-Second-Order	*k*_*2*_ *(g mg*^*-1*^ *min*^*− 1*^*)*	0.011
	*q*_*e*_ *(mg g*^*− 1*^*)*	66.66
	*R* ^*2*^	0.99
Elovich	*α (mg (g min)* ^−1^*)*	3158.5
	*β (g mg* ^*− 1*^ *)*	0.14
	*R* ^*2*^	0.94

#### Thermodynamic analysis

**Fig. 10:**
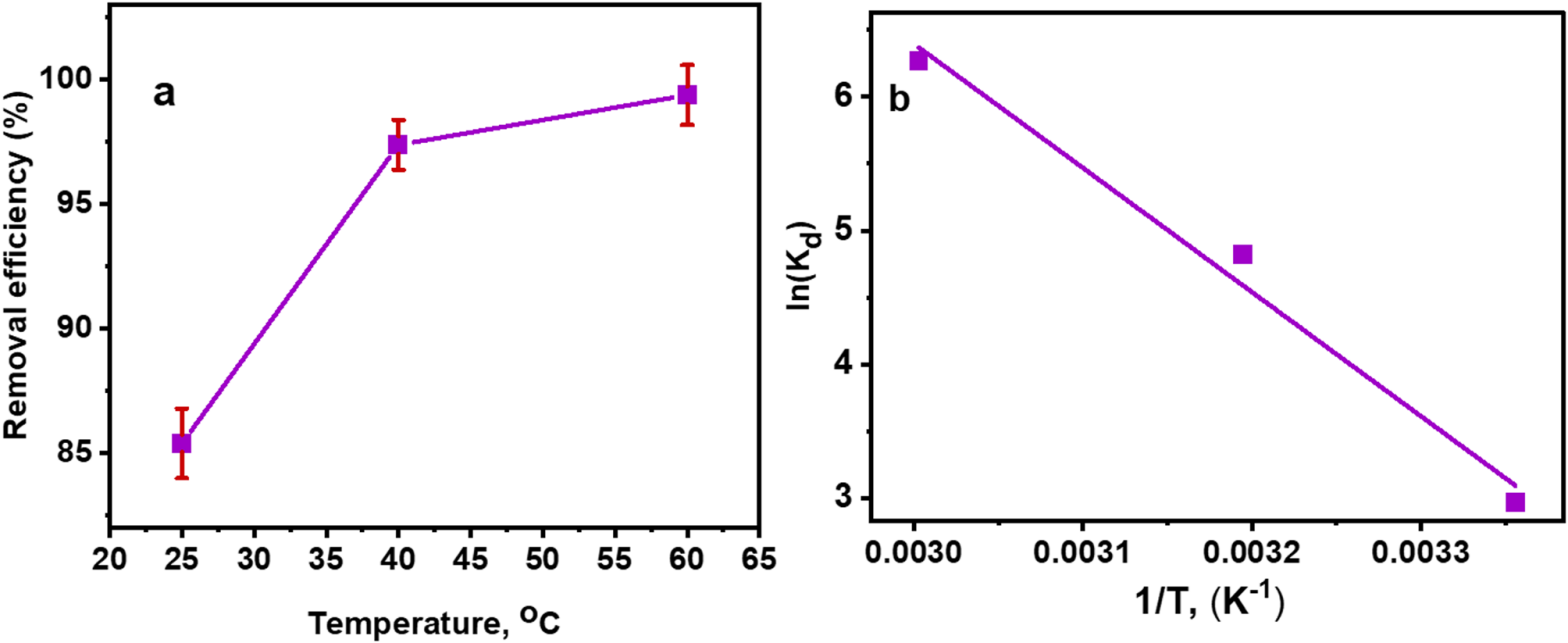
(**a**) Effect of temperature on CV removal efficiency by PNSAC-3 at initial concentration = 40 mg L^− 1^, adsorbent dose = 0.3 g L^− 1^, contact time = 20 min, and natural pH (5.6); error bars represent standard deviation (*n* = 3). (**b**) Van’t Hoff plot (ln K_d_ vs. 1/T) for CV adsorption onto PNSAC-3, yielding ΔH° = +77.24 kJ mol^− 1^, ΔS° = +284.91 J mol^− 1^ K^− 1^, R² = 0.986.

The effect of temperature on CV adsorption onto PNSAC-3 was investigated over the temperature range of 25–60 °C at an initial CV concentration of 40 mg L^− 1^, and the thermodynamic parameters were evaluated using the Van’t Hoff equation (Fig. 10). As shown in Fig. 10a, the removal efficiency increased from 85.37% at 25 °C to 97.37% at 40 °C, and reached 99.37% at 60 °C, indicating that higher temperatures favor the adsorption process and confirming its endothermic nature.

The thermodynamic parameters were determined from the linear Van’t Hoff plot (Fig. 10b), (R^2^ = 0.986) according to the following Eq.^81^:

12 $$\:\:\:\:\:\:\:\:\:\mathrm{ln}{K}_{d}=\frac{{\varDelta\:S}^{o}}{R}-\:\frac{{\varDelta\:H}^{o}}{RT}$$

13 $$\:\:\:\:\:\:\:\:\:\:\varDelta\:{G}^{o}=-RTln{K}_{d}$$

Where R is the universal gas constant (8.314 Jmol^−1^K^− 1^), T is the absolute temperature (K), and K_d_ is the apparent distribution coefficient, calculated as the ratio of the adsorption capacity (q_e_) to the equilibrium concentration (C_e_).

The calculated thermodynamic parameters are summarized in Table 6. The positive value of $\:\varDelta\:{H}^{o}$ (+ 77.24 kJmol^− 1^) confirms the endothermic nature of the adsorption process, indicating enhanced adsorption at elevated temperatures. Moreover, the positive $\:\varDelta\:{S}^{o}\:\:$value (+ 284.91 Jmol^− 1^ K^− 1^) suggests increased randomness at the solid-liquid interface during adsorption, possibly due to the displacement of water molecules from the adsorbent surface during CV uptake.

The negative $\:\varDelta\:{G}^{o}$ values obtained at all studied temperatures confirm the spontaneous and thermodynamically favorable nature of the adsorption process. Furthermore, the increasingly negative $\:\varDelta\:{G}^{o}$ values at higher temperatures indicate that adsorption becomes more favorable as temperature increases.

**Table 6:** Thermodynamic parameters for CV adsorption onto PNSAC-3 at different temperatures under optimized adsorption conditions (initial concentration = 40 mg L^− 1^, adsorbent dose = 0.3 g L^− 1^, contact time = 20 min, and natural pH).

Temperature (°C)	Temperature (K)	Removal (%)	K_d_ (L g^− 1^)	ln K_d_	$\:\varDelta\:{\boldsymbol{G}}^{\boldsymbol{o}}$ (kJmol^− 1^)	$\:\varDelta\:{\boldsymbol{H}}^{0}$ (kJmol^− 1^)	$\:\varDelta\:{\boldsymbol{S}}^{0}$ ( Jmol^− 1^ K^− 1^)
25	298	85.37	19.45	2.97	−7.35	+ 77.24	+ 284.91
40	313	97.37	122.30	4.81	−12.53		
60	333	99.37	524.80	6.26	−17.34		

#### Comparison with other adsorbents

The maximum Langmuir adsorption capacity of PNSAC-3 for the removal of CV was benchmarked against several recently reported adsorbents, as outlined in Table 7. PNSAC-3 exhibited competitive adsorption capacity compared with previously reported adsorbents, particularly when considering its operation at natural pH (5.6), a significantly lower adsorbent dosage (0.3 g L^− 1^), and a shorter equilibrium time (20 min). These results underscore the practical efficiency of PNSAC-3, as it achieves high pollutant removal without the need for intensive chemical or energy adjustments, making it an economically attractive candidate for real-world wastewater remediation.

**Table 7:** Comparison of CV dye adsorption capacity of PNSAC-3 and various activated carbons derived from diverse biomass sources.

Adsorbent	qmax (mg g⁻¹)	pH	C₀ (mg L^− 1^)	Dose (g L^− 1^)	Time (min)	Temp (°C)	%	Ref
Activated carbon from duckweed	61.39	6	30	0.5	60	30	95.74	^63^
Activated carbon from mixed orange peel and watermelon rind	137.80	10	200	1	35	25	91.6	^70^
Activated carbon from poultry litter	70.32	10	50	2.5	45	25	92.03	^82^
Carbon from Ricinus Communis Pericarp	48	6.8	25	0.5	60	30	100	^83^
Activated carbon from lemon wood	23.60	9	10	1.25	60–80	25	93.5	^84^
Activated carbon from reed straw	200.7	10	250	1	3.37	25	91.6	^85^
Activated carbons from rice husk	61.57	10.8	150	5	120	25	93.5	^86^
Activated Carbon from Sunflower Seed Pericarp	119.00	9.8	200	0.83	5.38	25	91	^87^
Activated carbon from food wastes (chicken bones and rice waste)	57.90	9	200	1.18	408	25	90.06	^88^
**PNSAC-3**	**145.35**	**5.6**	**20**	**0.3**	**20**	**25**	**98.9**	**This study**

#### Reusability

**Fig. 11:**
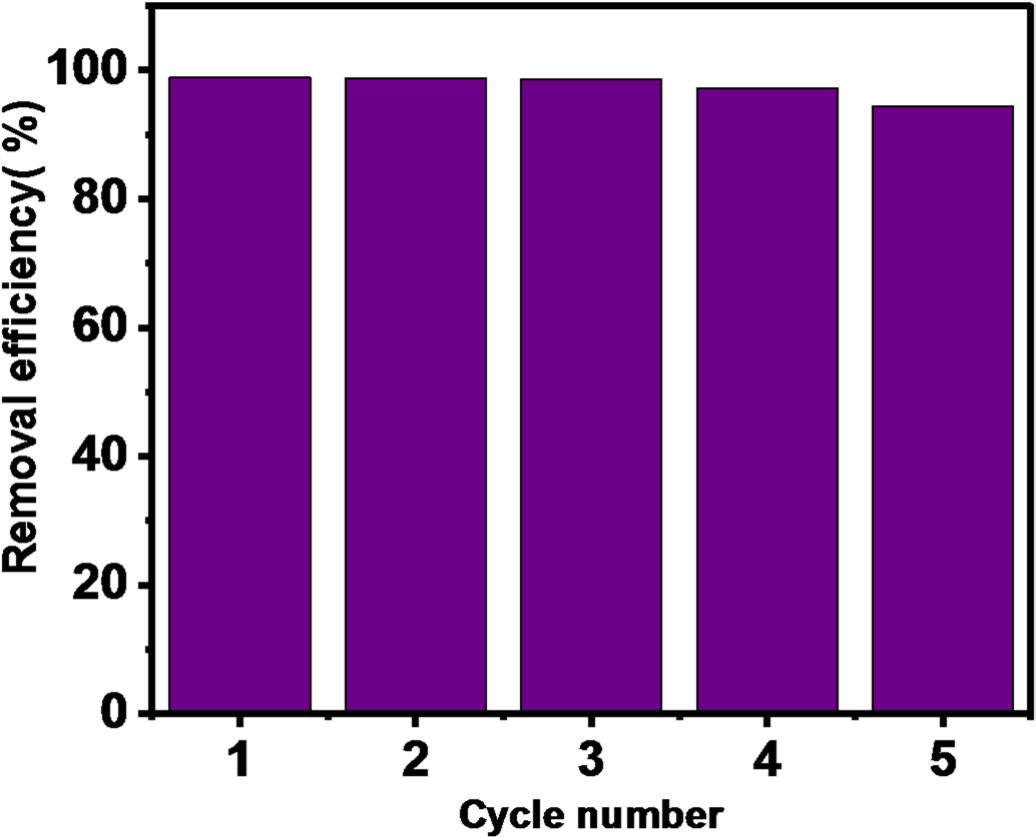
Reusability of PNSAC-3 over multiple adsorption–desorption cycles for CV removal (initial concentration = 20 mg L^− 1^, adsorbent dose = 0.3 g L^− 1^, pH = 5.6, temperature = 25 °C).

In wastewater remediation, high adsorbent reusability is a critical attribute from both economic and ecological standpoints, as it reduces the need for frequent adsorbent replacement and minimizes solid waste generation^89^. In this study, the reusable character of the PNSAC**-**3 adsorbent was tested by replicating the adsorption-desorption cycle five consecutive times under consistent operational parameters for CV removal. The data is shown in Fig. 11. The specific removal efficiencies observed for each cycle were 98.9%, 98.76%, 98.69%, 97.17%, and 94.4%, respectively. This minimal decline in performance, less than 5% after five cycles, demonstrates the excellent regeneration stability and robust reusability of PNSAC-3, indicating that most of the active adsorption sites remained effectively accessible throughout repeated adsorption–desorption cycles. From an economic perspective, such high reusability reduces the operational cost per treatment cycle and extends the service lifetime of the adsorbent, while minimizing the expenses associated with adsorbent replacement, adsorbent production, and energy consumption. Therefore, PNSAC-3 can be considered a cost-effective, highly efficient, and promising adsorbent for practical wastewater remediation applications.

## Conclusions

This study successfully demonstrated the synthesis of cost-effective and efficient activated carbons from peanut shells via phosphoric acid activation at 400 °C for the efficient removal of crystal violet (CV) dye from aqueous solutions. Among the synthesized materials, PNSAC-3 (PNS: H_3_PO_4_ weight ratio of 1:3) exhibited the highest performance, achieving 98.9% CV removal within 20 min under optimal conditions (adsorbent dose = 0.3 g L⁻¹, natural pH, 25 °C). Kinetic and equilibrium analyses indicated that the pseudo-second order and Langmuir models well described the adsorption process. The adsorption mechanism was attributed to the combined contribution of electrostatic attraction, π–π interactions, hydrogen bonding, and pore-filling effects. The relatively high adsorption capacity (145 mg g^− 1^), rapid adsorption rate, and excellent stability over five adsorption–desorption cycles demonstrate the strong potential of PNSAC-3 as an efficient and reusable adsorbent for cationic dye removal. Moreover, the use of abundant agricultural waste and relatively mild preparation conditions highlights the economic and environmental advantages of the developed material. Overall, the findings suggest that PNSAC-3 is a promising low-cost adsorbent for wastewater treatment applications. Future work should focus on large-scale implementation, optimization of regeneration strategies and acid recovery processes, and comprehensive evaluation of environmental impact to further enhance the practical applicability of the developed system.

## Supplementary Information

Below is the link to the electronic supplementary material.

Supplementary Material 1

## Data Availability

All data generated or analyzed during this study are included within the published article and its supplementary information files.
