# Supplementary material for: Toward sustainable activated carbon from peanut shells for efficient cationic dye removal: equilibrium, kinetics, and cost evaluation
Source: Sci Rep. 2026 Jun 26;16:19625. doi: 10.1038/s41598-026-58076-y (PMC13309566; doi:10.1038/s41598-026-58076-y)
Supplement: Supplementary file 1 — Supplementary Material 1 [file 41598_2026_58076_MOESM1_ESM.docx]

**Toward Sustainable Activated Carbon from Peanut Shells for Efficient Cationic Dye Removal: Equilibrium, Kinetics, and Cost Evaluation**

**Aya S. Mahmoud ^a,^*,Mohamed El Saied ^b^, Seham Ali Shaban ^b^, Ahmed O. Abo El Naga ^b,^***

^a^ Chemistry Department, Faculty of Women, Ain Shams University, Cairo, Egypt.

**^b^** Refining Division, Egyptian Petroleum Research Institute, 11727, Nasr City, Cairo, Egypt

**Corresponding author.**

Aya S. Mahmoud Email: [aya.sabry@women.asu.edu.eg](mailto:aya.sabry@women.asu.edu.eg)

Ahmed O. Abo El Naga Email: [amo_epri@yahoo.com](mailto:amo_epri@yahoo.com)

**Fig. S1.** X-ray diffraction (XRD) pattern of the raw PNS precursor

**Fig. S2.** N₂ adsorption-desorption isotherm of untreated biochar and pore size distribution

**
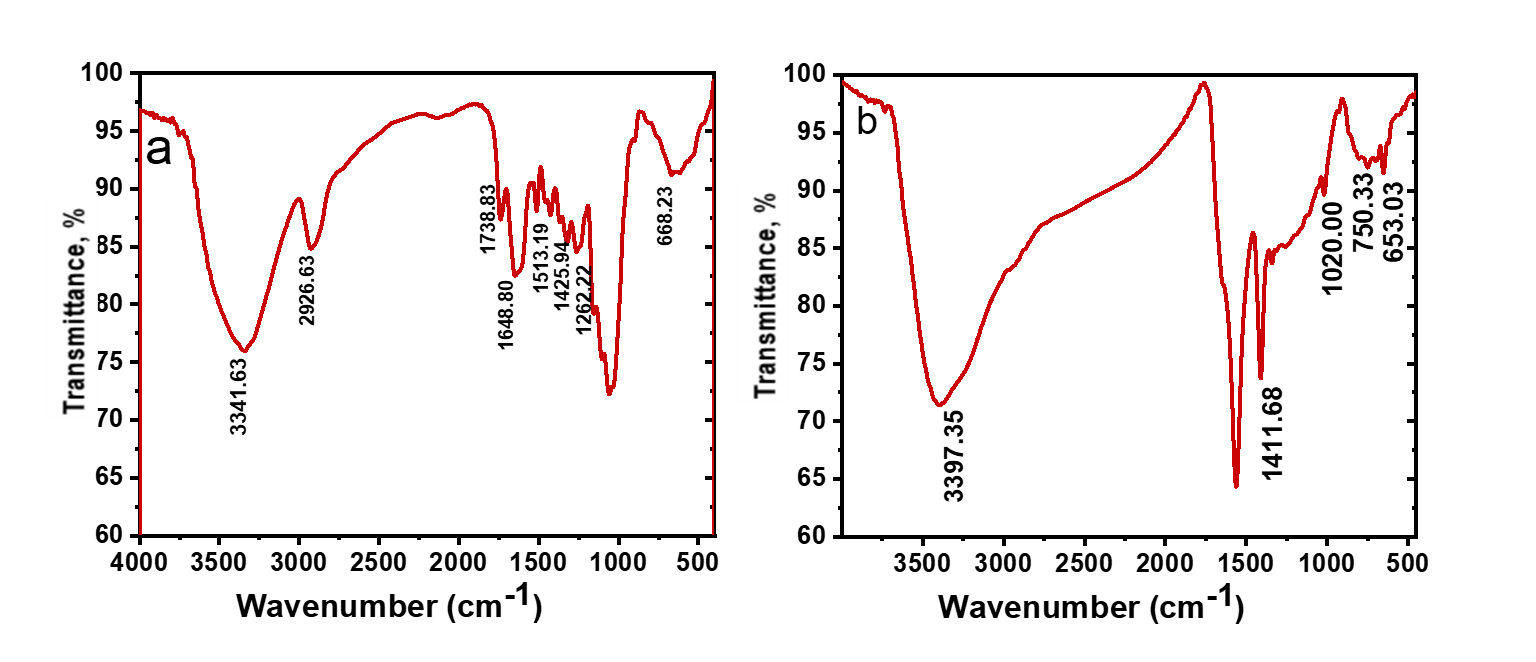
**

**Fig. S3.** FTIR spectra of (a) raw PNS precursor, and (b) untreated biochar (PNS carbonized at 400°C without H₃PO₄)





**Fig. S4.** SEM micrograph of raw PNS precursor (magnification: ×250, scale bar: 100 μm), showing a layered flaky morphology with smooth, non-porous surfaces characteristic of the intact lignocellulosic biomass structure.


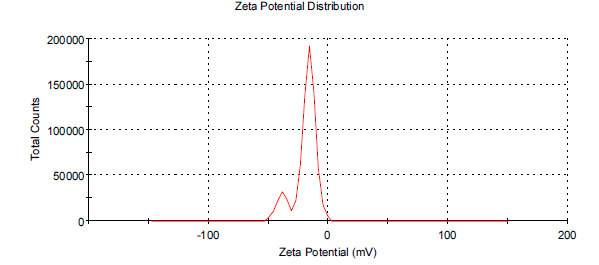


**Fig. S5.** Zeta potential distribution of PNSAC-3, showing a moderately negative surface charge (−19 mV) with slight heterogeneity.
